# Perceived Threat and Corroboration: Key Factors That Improve a Predictive Model of Trust in Internet-based Health Information and Advice

**DOI:** 10.2196/jmir.1821

**Published:** 2011-07-27

**Authors:** Peter R Harris, Elizabeth Sillence, Pam Briggs

**Affiliations:** ^1^Department of PsychologyUniversity of SheffieldSheffieldUnited Kingdom; ^2^Department of PsychologySchool of Life SciencesNorthumbria UniversityNewcastle upon TyneUnited Kingdom

**Keywords:** Internet, trust, e-health, threat, fear-appeal, social cognition models.

## Abstract

**Background:**

How do people decide which sites to use when seeking health advice online? We can assume, from related work in e-commerce, that general design factors known to affect trust in the site are important, but in this paper we also address the impact of factors specific to the health domain.

**Objective:**

The current study aimed to (1) assess the factorial structure of a general measure of Web trust, (2) model how the resultant factors predicted trust in, and readiness to act on, the advice found on health-related websites, and (3) test whether adding variables from social cognition models to capture elements of the response to threatening, online health-risk information enhanced the prediction of these outcomes.

**Methods:**

Participants were asked to recall a site they had used to search for health-related information and to think of that site when answering an online questionnaire. The questionnaire consisted of a general Web trust questionnaire plus items assessing appraisals of the site, including threat appraisals, information checking, and corroboration. It was promoted on the hungersite.com website. The URL was distributed via Yahoo and local print media. We assessed the factorial structure of the measures using principal components analysis and modeled how well they predicted the outcome measures using structural equation modeling (SEM) with EQS software.

**Results:**

We report an analysis of the responses of participants who searched for health advice for themselves (N = 561). Analysis of the general Web trust questionnaire revealed 4 factors: information quality, personalization, impartiality, and credible design. In the final SEM model, information quality and impartiality were direct predictors of trust. However, variables specific to eHealth (perceived threat, coping, and corroboration) added substantially to the ability of the model to predict variance in trust and readiness to act on advice on the site. The final model achieved a satisfactory fit: χ^2^_5_ = 10.8 (*P* = .21), comparative fit index = .99, root mean square error of approximation = .052. The model accounted for 66% of the variance in trust and 49% of the variance in readiness to act on the advice.

**Conclusions:**

Adding variables specific to eHealth enhanced the ability of a model of trust to predict trust and readiness to act on advice.

## Introduction

The Internet is an important source of health information and advice. Eight in 10 Internet users have looked online for health information [1] with young people in particular finding it to be a congenial source of health information and advice [2,3]. People use the Internet to help them with a variety of problems related to health, such as seeking reassurance or obtaining information before visiting the physician [4]. They may also turn to it after such visits if, for example, they feel they have not been given enough time or information to make sensible treatment choices [5] or find it difficult to recall elements of their consultations [6,7]. Yet the volume of available material and the unregulated nature of health information on the Internet pose potential problems for users. Indeed, investigations of eHealth material across a range of contexts typically conclude that quality is a problem (e.g., [8,9]). In the face of such variable quality, how do people decide which information to accept when seeking health information and advice?

Trust—“an expectancy held by an individual or group that the word, promise, verbal or written statement of another individual or group can be relied upon” (p. 651) [10]—is widely considered to be pivotal to this process. However, empirical research suggests that the strategies people use when deciding which Internet sites to trust are often suboptimal. For example, e-commerce consumers are influenced by potentially misleading cues, such as the look and feel of the site, trusting sites rated highly in visual appeal and mistrusting sites with poor visual design or with unprofessional errors [11-13]. In the health domain, users are also prone to reject high-quality medical sites because of poor visual appeal [14]; they also typically fail to check website authorship or read disclosure statements, despite suggesting these as important quality markers beforehand [15]. They also make prolific use of general search engines, thereby potentially exposing themselves to large numbers of poor-quality sites [16]. Perceptions of the motives underlying the site also appear to be important and may strongly influence the outcome. For instance, UK participants mistrusted the advice and information on websites openly sponsored by pharmaceutical or other commercial companies [14,17], even though such sites are often recommended by expert reviewers as providing the most accurate health information [18]. Likewise, very early in the selection process participants may reject high-credibility, high-quality sites if they do not appear to be aimed at “people like them” [17]. Users also have a broad view of what constitutes expertise, rating highly expertise displayed by patients and caregivers, as well as medical personnel, which may lead to disparities between the decisions the users make about site quality and those of expert reviewers [19].

To better understand the factors that determine trust, researchers have developed models of the process by which users form trust relationships online [14,16,20,21]. For example, Briggs et al [16] developed a 3-factor model, following analysis of a scale they developed to assess trust in e-commerce, in which trust was determined by 3 key predictors: source credibility (the extent to which the advice came from a knowledgeable, expert source), personalization (the extent to which the respondent felt involved in the advice process and the advice was tailored to them), and predictability (the extent to which the site appeared familiar and the advice met their expectations).

Such models provide a promising starting point for modeling trust in eHealth but, because they are typically either generic (eg, [20,21]) or have been developed to understand trust in another domain, such as e-commerce (eg, [16,22]), they are likely to require augmentation to improve their fit to trust in eHealth. People are not neutral processors of health-risk information [23]: those searching for an explanation for their symptoms naturally prefer one that is benign, and those taking tests to assess their chances of contracting some disease prefer to discover their chances are low [24,25]. In the health domain, that is, people often have strong initial expectations and show preferences for particular sorts of information, and these may shape their search strategies [26] and influence which sites they trust. They may respond differently to sites containing information and advice they find unwelcome or threatening than they respond to sites containing information they find congenial and comforting [23,27]. This distinctive element may not be captured by existing models of trust that have their origins elsewhere, such as in e-commerce. Accordingly, a key aim of this study was to examine whether adding variables from models designed to capture how people respond to health-risk information enhances the capacity of a general model of trust to account for (1) trust in advice presented on health websites and (2) readiness to act on that advice.

Researchers in health psychology have developed models to account for the ways in which people respond to health-risk information, including information the individual may find uncomfortable or threatening (eg, protection motivation theory [PMT] [28,29]; the extended parallel process model (EPPM) [30]) [31]. For example, in PMT and the EPPM, perceived threat—a product of the person’s appraisal of the harm that would occur as a result of the hazard (severity) and their personal susceptibility to it (vulnerability)—is a critical predictor of response to health information. The EPPM proposes that, as perceived threat increases, so does the individual’s appraisal of the extent to which they can take steps to control the hazard. Where they are persuaded that they can alleviate the hazard, they are motivated to do so [28,30]. Consequently, we assessed whether adding measures of threat, control, and coping affected perceptions of trust. Given the unregulated nature of the Internet, we also examined the possibility that the extent to which people are prepared to trust and accept unpalatable or threatening online information depends on whether it is corroborated. To test this, we included measures of the extent to which participants reported checking the information and finding it consistent with information they obtained elsewhere.

In the current study, we modeled trust and readiness to act on online health advice (our outcome measures). We did this in 2 steps: first, we assessed the factorial structure of a general measure of trust in online eHealth sources and modeled how well the factors predicted the outcomes. We did this by taking a model of online trust developed from data on e-commerce [16], supplementing it with items derived from our qualitative research on trust in eHealth [14,32], and testing it against a data set that covers a range of conditions, diseases, and health-related issues. Second, we assessed whether adding variables designed to capture elements of the response to health-risk information that is uncongenial (such as threat and control) and online (such as information checking and corroboration) enhanced the model’s predictive power.

## Method

### Participants

In total, 1902 participants completed the online questionnaire, which was promoted on the hungersite.com website. Visitors to the website could click on an advertising graphic and were transferred to the online survey. For each click on an advertisement on the hungersite page, a donation of US $0.05 is made to the UN World Food Programme. We had used the hungersite successfully in the past and chose it for this study because of its relatively broad international appeal and its focus on charitable donation. The URL for the questionnaire was also submitted to Yahoo and other search engines. A press release was also put out to local print media (eg, university newsletters and the local newspaper).

Incomplete questionnaires (mostly comprising 1 or 2 initial answers only) were removed (n = 415) and an internal consistency check was applied to the data to eliminate duplicate responders. This involved matching the respondent’s stated location with their Internet protocol (IP) address and led to a further 5 replies being removed, leaving a total of 1482 respondents. Of these, 1103 reported having used the Internet for health advice, of whom just over half (561, 51%) reported searching the Internet for health advice for themselves. This group (ie, those reporting searching for advice for themselves) formed the sample for the current paper (other respondents were not directed to the pages asking the threat appraisal questions). Of the sample, 402 (72%) were female and 92 (16%) were male (the remainder did not specify), and age ranged from under 18 to over 64 years and was spread quite evenly within this range (the modal age was 25–35 years old). There were no significant associations between searching for self and searching for other people in gender (n = 832, χ^2^_1_ = .35, *P* = .552), age (n = 832, χ^2^_5_ = 5.76, *P* = .330), highest education level (n = 832, χ^2^_3_ = 3.19, *P* = .364), or country of residence (n = 832, χ^2^_4_ = 9.31, *P* = .054; see Table 1).

**Table 1:** Background characteristics of participants and the health topics searched for

Participant characteristic		Frequency (%)	International Classification of Primary Care (where applicable)
**Gender**			
	Male	92 (16%)	
	Female	402 (72%)	
**Age (years)**			
	<18	21 (4%)	
	18–24	94 (19%)	
	25–35	129 (26%)	
	36–44	73 (15%)	
	45–54	90 (18%)	
	55–64	65 (13%)	
	>64	24 (5%)	
**Highest education level**			
	High school	70 (14%)	
	College	133 (27%)	
	University	151 (30%)	
	Postgraduate	142 (29%)	
**Country/region of residence**			
	United States	290 (59%)	
	Canada	37 (8%)	
	United Kingdom	84 (17%)	
	Western Europe	28 (6%)	
	Eastern Europe	8 (2%)	
	Australasia	17 (4%)	
	Central/South America	9 (2%)	
	Middle East	4 (<1%)	
	Africa	1 (<1%)	
	Japan	5 (1%)	
	Other	5 (1%)	
**Internet use (years)**			
	1–2	9 (2%)	
	3–5	66 (14%)	
	6–9	198 (41.2%)	
	10–14	169 (35.1%)	
	15–19	30 (6%)	
	≥20	9 (2%)	
**Health topic**			
	Allergies	19 (3%)	A92
	Arthritis	23 (4%)	L88
	Alternative health	42 (7%)	
	Cancer	17 (3%)	Type of cancer not specified
	Children’s health	8 (1%)	Not specified
	Diabetes	10 (2%)	T89
	Diet	43 (8%)	
	Depression	20 (4%)	P76
	Fitness	37 (7%)	
	Heart disease	19 (3%)	K71
	Hypertension	8 (1%)	K86
	Men’s health	19 (3%)	
	Mental health (excluding depression)	12 (2%)	
	Migraine	9 (2%)	N89
	Women’s health	98 (17%)	
	Sexually transmitted diseases	20 (4%)	
	Thyroid problems	17 (3%)	T85; T86
**Other**		140	
	Skin conditions (eczema, psoriasis, rosacea)	9 (2%)	S87; S91; S90
	Fibromyalgia	5 (<1%)	L18
	Fertility issues, pregnancy, miscarriage	5 (<1%)	W15; W78; W82
	Influenza, pneumonia, colds	5 (<1%)	R80; R81; R74
	Parkinson disease, multiple sclerosis, Alzheimer disease	4 (<1%)	N87; N86; P70
	Anorexia	3 (<1%)	P86
	Back pain	3 (<1%)	L02
	Cold sores	2 (<1%)	S71
	Heartburn	2 (<1%)	D03
	Mumps	1 (<1%)	D71
	High cholesterol	1 (<1%)	T93

### Procedure

On the first page of the online questionnaire, participants were asked whether they had sought advice online about health. Those responding yes were taken to the subsequent screens of questions, including questions about the site they had previously used and their reasons for searching online, as well as demographic information (gender, age, educational attainment, country of residence, and length of Internet use) and the predictor and outcome measures. We report data from those participants answering yes to either of 2 key questions: “Last time you searched online for health advice were you trying to find out whether you might already have a particular disease/condition?” and “Last time you searched online for health advice were you searching for information/advice about the chances of you getting or preventing yourself from getting a particular disease in the future?” Participants were then asked to “Think about any one site that you visited during that search” and to answer the remaining questions with respect to that site—that is, “Thinking about the information or advice on the site please rate your agreement with the following statements.”

### Measurements

Once participants had responded to the above statements, the following measures were taken. Except where indicated, responses were given on a 5-point scale (1 = *strongly disagree* to 5 = *strongly agree*).

#### General Web Trust Questionnaire

The first measure contained the items designed to assess various aspects of trust in online sources derived from the 18-item trust questionnaire developed by Briggs et al [16] supplemented by 6 items derived from the qualitative research on eHealth conducted by Sillence and colleagues [14,32]. (The full set of items can be found in Table 2.)

#### Threat appraisal

Consistent with measurement of threat in PMT and the EPPM, threat appraisal was measured by combining 2 items we developed, one to assess susceptibility, “the site said my chances of having/getting the disease/condition were” (1 = *very low* to 5 = *very high*), and one to assess severity, “the site said my consequences of having the disease or condition were” (1 = *not at all severe* to 5 = *extremely severe*).

#### Reactions to the site

Affective and cognitive reactions to the site were assessed by 8 items. Following the stem “Looking at this site made me feel...” came the variables worried, reassured, at risk, confused, anxious about the risks, optimistic, in control, and able to cope*.* Responses were given on a 6-point scale with the following labels: 1 = *less,* 2 = *slightly less,* 3 = *no different,* 4 = *slightly more,* and 5 = *more*, plus *not applicable*.

#### Information checking and corroboration

In each case, two items measured (1) the extent to which participants checked other sources of information in addition to the website (“I checked other websites” and “I checked other sources”; *checked*, *r* = .52, *P* < .001), and (2) how consistent the advice was with these other sources (“I found the advice consistent with other websites” and “I found the advice consistent with other sources”; *corroboration*, *r* = .74, *P* < .001).

#### Outcome measures

The two outcome measures were *trust,* “I trusted the site,” and *readiness to act on the advice* the participants found on the site, “I intended to act upon the advice.” These were developed for the study.

## Results

Participants reported a wide range of diseases and conditions, from cancer, depression, and Alzheimer disease to thyroid problems, allergies, and mumps (see Table 1). On average, participants reported moderate levels of threat (mean 3.63 [SD 1.11]).

To retain the separate identities of the general trust questionnaire and the measure developed here of specific reactions to the site, these measures were factor analyzed separately using principal component analysis with varimax rotation. Analysis of the general Web trust questionnaire revealed 4 factors accounting for 55.6% of the variance (see Table 2). The number of factors was determined by consulting the scree plot and with reference to Kaiser’s eigenvalue > 1 criterion. Factor 1 (alpha = .85), labeled *access to quality information*, brought together items mainly describing ease of use and access to high-quality information. Factor 2 (alpha = .86), labeled *personalization*, brought together items mainly describing the importance of tailored information and the ability to interact with “like-minded people” on the website. Factor 3 (alpha = .74), labeled *perceived impartiality,* brought together items describing the extent to which the advice on the website appeared impartial and objective. Factor 4 (alpha = .70), labeled *credible design,* brought together items describing the extent to which the site had credible design features.

**Table 2:** Four factors on the general Web trust questionnaire and items with loading on the relevant factor

Item number		Items and factor loading
**Access to quality information**		
	6	The language on the site made it easy to understand (.77)^a^
	7	The site helped me understand the issue better (.76)
	5	The site was easy to use (.75)
	1	The site told me most of what I needed to know (.67)
	8	The layout was consistent with other sites (.50)
	2	The advice appeared to be prepared by an expert (.49)
	12	The advice seemed to be offered in my best interests (.49)
	11	The advice came from a knowledgeable source (.48)
**Personalization**		
	19	The site gave me a sense of being part of a community (.81)^a^
	10	I was able to contribute to content on the site (.76)^a^
	18	I felt involved in the way the site tried to find appropriate advice (.73)
	17	It felt like the advice was tailored to me personally (.71)
	14	The site contained contributions from like-minded people (.70)
	16	There were opportunities to interact with other people on the site (.70)^a^
	9	I identified with the site (.49)^a^
	22	The reasoning behind the advice was explained to me (.46)
**Perceived impartiality**		
	21	The advice appeared to be impartial and independent. (.78)
	20	The site was free from advertisements (.73)^a^
	13	The advice seemed objective (ie, no hidden agenda) (.61)
	23	The advice seemed credible (.56)
**Credible design**		
	4	The site was owned by a well-known organization (.76)
	15	The site featured familiar logos (.72)
	3	The site had a professional design (.60)
	24	The site had an attractive design (.52)

^a^ Item derived from Sillence and colleagues [14,31].

Analysis of the 8 items assessing reactions to the site revealed 2 factors: factor 1, which we labeled *coping* (alpha = .87), comprised the control, coping, and optimism items; factor 2, which we labeled *worry* (alpha = .77), comprised the worried, anxious, and at-risk items. The remaining items (confused and reassured) did not load significantly on either factor, so were entered separately in analyses.

To assess how scores on each of the above factors contributed to the prediction of trust and readiness to act on the advice, mean scores on each factor for each participant were calculated and entered into models as described below. Descriptive statistics for each variable in the final model, together with their intercorrelations, are presented in Table 3. 

**Table 3:** Descriptive statistics and zero-order correlations between the measures^a^

		Information quality	Personalization	Impartiality	Credible design	Coping	Threat	Corroboration	Trust	Mean	SD
Information quality										3.95	0.64
Personalization		.43**								2.72	0.90
Perceived impartiality		.61**	.29**							3.72	0.82
Credible design		.54**	.30**	.28**						3.41	0.84
Coping		.41**	.33**	.30**	.18**					3.73	1.02
Threat		.18**	.09*	.10*	.07	.66**				3.63	1.11
Corroboration		.23**	.08	.17**	.09*	.19**	.57**			3.44	1.76
**Outcomes**	
	Trust	.39**	.17**	.33**	.19**	.27**	.66**	.71**		3.68	1.58
	Readiness to act	.24**	.17**	.17**	.08	.29**	.55**	.62**	.63**	3.30	1.88

^a^ The range of each scale was the same as the scale points described in the procedure

*P < .05;

**P < .01.

The data were analyzed next using structural equation modeling with EQS (version 6.1; Multivariate Software Inc, Encino, CA, USA). The fit of the models was evaluated using chi-square, the comparative fit index (CFI), and the root mean square error of approximation (RMSEA). Satisfactory fit of the model is obtained when chi-square is nonsignificant, CFI is >.90, and RMSEA is <.08 [33]. Path coefficients and *R* ^2^ values were also inspected in evaluating the predictive power of the models.

EQS was first used to test a model that included all paths from the 4 factors of the general Web trust questionnaire to the outcomes, trust and readiness to act on the advice. The extent to which the variables additional to these 4 factors had unmediated effects on the outcomes was then examined by introducing, in turn, a direct path between each predictor variable and each outcome variable. Alternative models were compared using the different fit indices and the extent to which they explained variance in trust and readiness to act (*R* ^2^).

**Figure 1:**
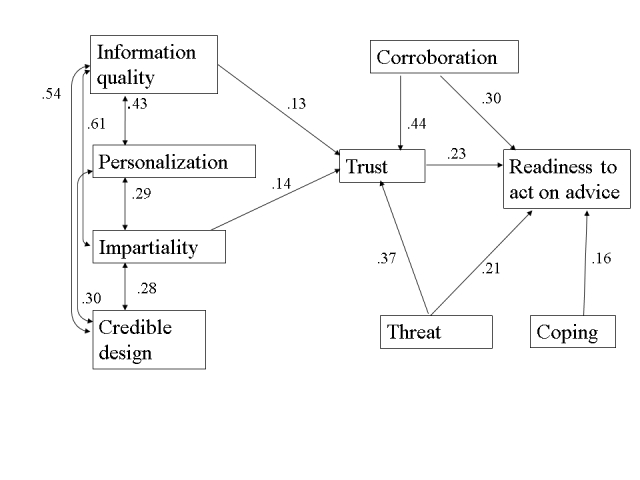
The final model, showing the significant path coefficients (P < .05)

The initial model accounted for 15% of the variance in trust and 39% of the variance in readiness to act and had a poor fit (χ^2^_4_ = 11.3, *P* = .02, CFI = .99, RMSEA = .057); as predicted, adding *threat* improved the fit of the model, as did adding *coping* and *corroboration*. Adding the remaining variables (ie, *worry*, *checked, confused, reassured*) did not improve fit. The added variables produced no substantive changes in other paths in the model. The fit of the final model was good: chi-square was not significant (χ^2^_5_ = 10.8, *P* = .21), the other measures of fit indicated a good fit (CFI = .99, RMSEA = .052), and the model accounted for 66% of the variance in trust (*R* ^2^ = .66) and 49% of the variance in readiness to act on the advice (*R* ^2^ = .49). The final model, with its significant pathways (*P* < .05), is displayed in Figure 1. Descriptive statistics and intercorrelations for the measures not included in the final model are in Multimedia Appendix 1.

## Discussion

The current study aimed to (1) assess the factorial structure of a general measure of Web trust, (2) model how the resultant factors predicted trust in, and readiness to act on, the advice found on health-related websites, and (3) test whether adding variables to capture elements of the response to uncongenial, online health-risk information enhanced the prediction of these outcomes. As predicted, incorporating the latter variables added to the ability of the model to predict variance in both trust and expressed readiness to act on advice. The final model accounted for substantial amounts of variance in both outcome measures. Four factors emerged from analysis of the general Web trust questionnaire. However, a key feature of our final model is that only 2 of these, impartiality and information quality, had direct effects on trust in health-related websites; the effects of the other 2 factors, personalization and credible design, were indirect and mediated through impartiality and information quality. This is consistent with findings from the earlier, qualitative phase of the current research program, which also signaled that, in the health domain, seeking high-quality, independent advice might be critical [17]. It is notable that in previous research in other domains impartiality has emerged as a predictor but not occupied the pivotal role it has here [16]. Indeed, impartiality is not necessarily expected in such domains as e-commerce, although when it is encountered it is valued [13]. The importance of credibility through impartiality here is also consistent with the basis of trust in patient–physician relationships, in which there is an assumption that physicians will act in the best interests of the patient [34].

Clearly, elements of the general trust component of the current model correspond with those in other trust models [21], suggesting that researchers are isolating the core general features of trust across a range of domains, from eHealth through e-commerce to leisure [20,22,35,36]. What differs appears to be the relative importance of certain factors in each domain and at different phases of the trust process [16,17]. However, it is notable that the initial model, which contained only the general Web trust factors, formed a model with relatively poor fit to the data, supporting the decision to search for additional variables to enhance fit to a specific domain (in this case, eHealth).

Both threat and corroboration contributed to the prediction of trust, with direct positive relationships in each case. According to these data, therefore, people are prepared to trust sites that tell them things they can verify elsewhere and sites that tell them things they would prefer not to hear. It is interesting to find that they do not, therefore, appear to let unwelcome news interfere with the process of assessing trust. Research needs next to address moderators of this relationship. For example, do those high in need for closure [37] or low in tolerance for uncertainty [38] show less readiness to trust sites containing high-quality but uncongenial information? As a variable, trust may also have potential to help clarify the different relationships found in previous research between threat and message acceptance, where meta-analyses have, for example, found a linear relationship between fear and intentions [30], but studies have also shown that the most threatened groups often also show the least acceptance (see [23,27] for recent reviews).

The absence of a relationship between coping and trust is also worthy of note. Just as one might have expected people to want to trust sites telling them things about vulnerability and disease severity they found congenial, one might have expected people also to want to trust sites telling them positive things about their ability to control diseases or otherwise boost their sense of efficacy and coping; yet here this was not the case. Indeed, in many ways the current findings illustrate that trust is not simply likeability: people appear to be prepared to trust sites containing information they dislike. In developing trust perceptions, our findings suggest that people may be more prepared to accept information they dislike than basic design features they dislike [32].

Encountering a site that increased one’s perceptions of being able to cope, along with threat and corroboration, contributed independently and positively to the individual’s readiness to act on the advice the site provided. Indeed, while trust has been shown in this study to predict readiness to respond to advice, and to partially mediate the effects of threat and corroboration in the process, in the model outlined here it is only one of several independent contributors to readiness to act. The benefits from adding threat-related and other variables suggest that our understanding of the process by which trust perceptions are translated into relevant behavior is likely to be enhanced by further integration with relevant social cognition models of behavior, such as the PMT or the EPPM. The model outlined here is also one of the few models of trust to explicitly account for the perceived costs of undertaking a transaction (see [36] for another). Notions of vulnerability, cost, or perceived risk have been argued to be effective in improving the predictive value of trust models in a variety of contexts [39].

The study has a number of limitations. It is not possible to assess the extent to which retrospective biases (such as a schema for rationality) may be contributing to the picture presented. Nevertheless, with such a large sample size and range of diseases and medical conditions (see Table 1), it is clear that this rational model comprises a significant element of the picture people tell the world (and perhaps also themselves) about their responses to health information they encounter on the Internet. Unfortunately, it was not possible to test whether those who started to complete the online questionnaire, but quickly dropped out, differed in key ways from those who persisted. It remains possible, therefore, that the current sample may be unrepresentative. However, the sample tested here (those searching for information for themselves) did not differ from the other group of respondents who also completed the survey (those searching for information for someone else) in terms of the key variables reported in Table 1, which offers some reassurance about their representativeness. It would also be useful to assess in future research of this kind the individual respondent’s health status, to establish whether this moderates responses. Likewise, while it is clear from the research reported here that the inclusion of threat-related variables in a model of trust helps predict readiness to act on the advice, it is also known that there is often a gap between intentions and behavior. A complete picture needs to dovetail the processes modeled here with those known to affect the process of translating intentions into behavior [31,40]. What determines impartiality perceptions is also likely to vary between cultures. For example, in societies in which commercial interests are more readily linked to health care provision than in the United Kingdom, the predictors of impartiality may differ. The role of such factors may also be the focus of valuable future research.

The final model supports the hypothesis that people will seek to validate what they find on websites against other sources of information. If they find that information is corroborated elsewhere, this boosts trust in the website. Given the relative novelty of websites as sources of health information, this process can be seen as one of calibrating the credibility of these novel sources against more tried and tested ones. Over time, as people become more experienced and adept at assessing the credibility of Internet sources, we would expect this checking process to become less important.

Finally, for website developers, the current data contain some useful pointers. Four key design factors (information quality, personalization, perceived impartiality, and credible design) have an important (albeit moderated) role to play in influencing trust and the subsequent decision to act on the advice given.

## Multimedia Appendix 1

Zero-order correlations among the measures not included in the final model together with their descriptive statistics

## Abbreviations

CFI: comparative fit index
EPPM: extended parallel process model
IP: Internet protocol
PMT: protection motivation theory
RMSEA: root mean square error of approximation

## References

[1] Fox S (2011). Peer-to-peer healthcare.

[2] Berger M, Wagner TH, Baker LC (2005). Internet use and stigmatized illness. Soc Sci Med.

[3] Klein JD, Wilson KM (2002). Delivering quality care: adolescents' discussion of health risks with their providers. J Adolesc Health.

[4] Rozmovits L, Ziebland S (2004). What do patients with prostate or breast cancer want from an Internet site? A qualitative study of information needs. Patient Educ Couns.

[5] Carvel J (2005). Lack of information worries NHS patients. The Guardian.

[6] Kessels RP (2003). Patients' memory for medical information. J R Soc Med.

[7] Jansen J, Butow PN, van Weert JCM, van Dulmen S (2008). Does age really matter? Recall of information presented to newly referred patients with cancer. J Clin Oncol.

[8] Fox S, Rainie L (2000). Pew Internet and American Life Project.

[9] Eysenbach G, Powell J, Kuss O, Sa ER (2002). Empirical studies assessing the quality of health information for consumers on the world wide web: a systematic review. JAMA.

[10] Rotter JB (1967). A new scale for the measurement of interpersonal trust. J Pers.

[11] Fogg BJ, Marshall J, Laraki O (2001). What makes web sites credible? A report on a large quantitative study. Human Factors & Computing Systems Proceedings, 2001 Mar 31-Apr 5, Seattle.

[12] Kim J, Moon JY (1998). Designing towards emotional usability in customer interfaces: trustworthiness of cyber-banking system interfaces. Interacting with Computers.

[13] Standford J, Tauber E, Fogg BJ, Marable L Consumer Reports WebWatch.

[14] Sillence E, Briggs P, Harris P, Fishwick L (2006). A framework for understanding trust factors in web-based health advice. Int J Hum Comput Stud.

[15] Eysenbach G, Köhler C (2002). How do consumers search for and appraise health information on the world wide web? Qualitative study using focus groups, usability tests, and in-depth interviews. BMJ.

[16] Briggs P, Burford B, De Angeli A, Lynch P (2002). Trust in online advice. Soc Sci Comput Rev.

[17] Sillence E, Briggs P, Harris PR, Fishwick L (2007). How do patients evaluate and make use of online health information?. Soc Sci Med.

[18] Reed M, Anderson C (2002). Evaluation of patient information Internet web sites about menopause and hormone replacement therapy. Maturitas.

[19] Sillence E, Briggs P, Herxheimer A (2004). Personal experiences matter: what patients think about hypertension information online. He@lth Infom Internet.

[20] Corritore CL, Kracher B, Wiedenbeck S (2003). On-line trust: concepts, evolving themes, a model. Int J Hum Comput Stud.

[21] Wang YD, Emurain HH (2005). An overview of online trust: concepts, elements and implications. Comput Hum Behav.

[22] Egger FN, Helander M, Hkalid  HM, Tham MP (2001). Affective design of e-commerce user interfaces: how to maximise perceived trustworthiness. Proceedings of CAHD2001.

[23] Good A, Abraham C (2007). Measuring defensive responses to threatening messages: a meta-analysis of measures. Health Psychol Rev.

[24] Renner B (2004). Biased reasoning: adaptive responses to health risk feedback. Pers Soc Psychol Bull.

[25] Ditto PH, Lopez DF (1992). Motivated skepticism: use of differential decision criteria for preferred and nonpreferred conclusions. J Personal Soc Psychol.

[26] Joinson AN, Banyard P (2002). Psychological aspects of information seeking on the Internet. Aslib Proc.

[27] Harris PR, Epton T (2010). The impact of self-affirmation on health-related cognition and health behaviour: issues and prospects. Soc Personal Psychol Compass.

[28] Rogers RW, Cacioppo BL, Petty LL (1983). Cognitive and physiological processes in fear appeals and attitude change: a revised theory of protection motivation. Social Psychophysiology: A Sourcebook.

[29] Rogers RW, Prentice-Dunn S, Gochman DS (1997). Protection motivation theory. Handbook of Health Behavior Research.

[30] Witte K (1992). Putting the fear back into fear appeals: the extended parallel process model. Commun Monogr.

[31] Sheeran P, Milne S, Webb TL, Gollwitzer PM, Conner M, Norman P (2005). Implementation intentions and health behaviour. Predicting Health Behaviour: Research and Practice with Social Cognition Models, 2nd ed.

[32] Sillence E, Briggs P, Fishwick L, Harris P (2004). Trust and mistrust of online health sites. Chi 2004: Connect: Conference Proceedings: April 24-29, Vienna, Austria: Conference on Human Factors in Computing Systems: Vienn (Chi Letters).

[33] Marsh H, Balla J, Hau KT, Marcoulides GA, Schumacker RE (1996). An evaluation of incremental fit indices: a clarification of mathematical and empirical properties. Advanced Structural Equation Modeling: Issues and Techniques.

[34] Hall MA, Zheng B, Dugan E, Camacho F, Kidd KE, Mishra A, Balkrishnan R (2002). Measuring patients' trust in their primary care providers. Med Care Res Rev.

[35] Egger FN (2000). “Trust me, I’m an online vendor”: towards a model of trust for e-commerce system design. CHI Extended Abstracts on Human Factors in Computing Systems.

[36] Lee J, Kim J, Moon JY (2000). What makes Internet users visit cyber stores again? Key design factors for customer loyalty. CHI 2000, The Future is Here: CHI 2000 Conference proceedings: Conference on Human Factors in Computing Systems.

[37] Kruglanski AW, Webster DM, Klem A (1993). Motivated resistance and openness to persuasion in the presence or absence of prior information. J Pers Soc Psychol.

[38] O'Neill SC, DeMarco T, Peshkin BN, Rogers S, Rispoli J, Brown K, Valdimarsdottir H, Schwartz MD (2006). Tolerance for uncertainty and perceived risk among women receiving uninformative BRCA1/2 test results. Am J Med Genet C Semin Med Genet.

[39] Patrick AS, Briggs P, Marsh S, Cranor L, Garfinkel S (2005). Designing systems that people will trust. Security and Usability: Designing Secure Systems That People Can Use.

[40] Schwarzer R, Schwarzer R (1992). Self-efficacy in the adoption and maintenance of health behaviors: theoretical approaches and a new model. Self-Efficacy: Thought Control Of Action.

