# Supplementary material for: Perceived Threat and Corroboration: Key Factors That Improve a Predictive Model of Trust in Internet-based Health Information and Advice
Source: J Med Internet Res. 2011 Jul 27;13(3):e51. doi: 10.2196/jmir.1821 (PMC3222185; doi:10.2196/jmir.1821)
Supplement: Supplementary file 1 [file jmir_v13i3e51_app1.pdf]

Multimedia Appendix 1: *Zero-order Correlations among the measures not included in the final model together with their descriptive statistics*

	**Worry**	**Confused**	**Reassured**	**Mean**	**Standard deviation**
**Worry**				2.53	0.86
**Confused**	.54[b]			2.08	1.04
**Reassured**	-.50[b]	-.43[b]		3.77	0.98
**Checked**	.02	-.08	.11[a]	4.00	1.09
**Information quality**	-.20[b]	-.46[b]	.32[b]	3.95	0.64
**Personalization**	-.15[b]	-.24[b]	.18[b]	2.72	0.90
**Perceived impartiality**	-.12[b]	-.33[b]	.18[b]	3.72	0.82
**Credible design**	-.10[a]	-.20[b]	.18[b]	3.41	0.84
**Coping**	-.51[b]	-.52[b]	.54[b]	3.73	1.02
**Threat**	-.00	-.20[b]	.12[a]	3.63	1.11
**Corroboration**	-.14[b]	-.24[b]	.26[b]	3.44	1.76
**Outcomes**					
**Trust**	-.25[b]	-.47[b]	.33[b]	3.68	1.58
**Readiness to act**	-.22[b]	-.37[b]	.30[b]	3.30	1.88

[a] $p<.05$. [b] $p<.01$.

The range of each scale was the same as the scale points described in the procedure.
